# Targeted Activation of HNF4α by AMPK Inhibits Apoptosis and Ameliorates Neurological Injury Caused by Cardiac Arrest in Rats

**DOI:** 10.1007/s11064-023-03957-1

**Published:** 2023-06-20

**Authors:** Haohong Zhan, Qiang Zhang, Chenyu Zhang, Jingge Cheng, Yilin Yang, Cong Liu, Shuhao Li, Chuyue Wang, Junqin Yang, Hanmei Ge, Dawang Zhou, Bo Li, Hongyan Wei, Chunlin Hu

**Affiliations:** 1 Department of Emergency Medicine, The First Affiliated Hospital, Sun Yat-sen University, Guangzhou, 510080 China; 2 Department of Emergency Medicine, The Seventh Affiliated Hospital, Sun Yat-sen University, Shenzhen, 518107 China; 3 Department of Critical Care Medicine, The First Affiliated Hospital of Sun Yat-sen University, Guangzhou, 510080 China; 4 National Health Council (NHC) Key Laboratory of Assisted Circulation, Guangzhou, 510080 China

**Keywords:** AICAR, AMPK, HNF4α, Apoptosis, Cardiopulmonary resuscitation, Neuroprotection

## Abstract

Previous studies have shown that AMPK plays an important role in cerebral ischemia–reperfusion injury by participating in apoptosis, but the exact mechanism and target of action remains unclear. This study aimed to investigate the protective mechanism of AMPK activation on brain injury secondary to cardiac arrest. HE, Nills and TUNEL assays were used to evaluate neuronal damage and apoptosis. The relationships between AMPK, HNF4α and apoptotic genes were verified by ChIP-seq, dual-luciferase and WB assays. The results showed that AMPK improved the 7-day memory function of rats, and reduced neuronal cell injury and apoptosis in the hippocampal CA1 region after ROSC, while the use of HNF4α inhibitor weakened the protective effect of AMPK. Further research found that AMPK positively regulated the expression of HNF4α, and AMPK could promote the expression of Bcl-2 and inhibit the expression of Bax and Cleaved-Caspase 3. In vitro experiments showed that AMPK ameliorated neuronal injury by inhibiting apoptosis through the activation of HNF4α. Combined with ChIP-seq, JASPAR analysis and Dual-luciferase assay, the binding site of HNF4α to the upstream promoter of Bcl-2 was found. Taken together, AMPK attenuates brain injury after CA by activating HNF4α to target Bcl-2 to inhibit apoptosis.

**Supplementary Information:**

The online version contains supplementary material available at 10.1007/s11064-023-03957-1.

## Introduction

Cardiac arrest (CA) is one of the major causes of death and disability. With the continuous development and improvement of cardiopulmonary resuscitation (CPR) technology, the rate of return of spontaneous circulation (ROSC) has reached 20–40%, but the overall survival rate is still very low [1]. The brain is extremely sensitive to ischemia, and post-cardiac arrest brain injury (PCABI) is prone to occur during resuscitation of cardiac arrest, which is also the leading cause of death in resuscitated patients, accounting for approximately 60% of the deaths after ROSC [2, 3]. Due to the poor prognosis and complicated mechanism of neurological function in patients with CA, drug intervention to improve it is still lacking.

CA/CPR is a process of systemic ischaemia‒reperfusion, in which a cellular energy sensor, AMP-activated protein kinase (AMPK), is activated in response to a variety of conditions that deplete cellular energy levels, such glucose deprivation, hypoxia and exposure to toxins that inhibit the mitochondrial respiratory chain complex [4, 5]. AMPK is a serine/threonine protein kinase complex consisting of a catalytic α-subunit (α1 and α2), a scaffolding β-subunit (β1 and β2) and a regulatory γ-subunit (γ1, γ2 and γ3) [6]. In higher eukaryotes, AMPK has acquired the capacity to sense the amount of energy available in the cell by directly binding adenine nucleotides [7]. Upon changes in energy availability and thus changes in the ADP-to-ATP or AMP-to-ATP ratio, AMPK is activated by an allosteric mechanism that stimulates its kinase activity. Once activated, AMPK redirects metabolism towards increased catabolism and decreased anabolism through the phosphorylation of key proteins in multiple pathways, including glycolysis [8, 9], lipid homeostasis [10, 11], and mitochondrial homeostasis [12, 13]. Previous studies have shown that AMPK is involved in neuroprotection after cerebral ischaemia [14]. The AMPK activator 5-aminidazole-4-carboxamide riboside (AICAR) has been shown to inhibit apoptosis and alleviate organ injury in rodents [15, 16].

In addition to directly regulating key enzymes, AMPK also controls metabolism at the transcriptional level by phosphorylating sterol regulatory element-binding protein 1 (SREBP1) [17], carbohydrate-responsive element-binding protein (ChREBP) [18], hepatocyte nuclear factor 4α (HNF4α) [19] and transcription factor EB (TFEB) [20], which are key transcriptional regulators of lipid and glucose metabolism. HNF4α is a highly conserved nuclear transcription factor that binds DNA as a homodimer [21]. Multiple HNF4α isoforms exist in humans and are suggested to have different physiological roles in the development and transcriptional regulation of target genes. Previous studies have found that HNF4α is a key regulator of a number of genes involved in glucose, cholesterol and fatty acid metabolism [22]. The data point to HNF4A as a major hypoxia-responsive transcription factor in the cerebellum, and most hypoxia-regulated transcripts have at least one hepatic nuclear receptor 4α (HNF4α) binding site [23]. Recently, some studies demonstrated that HNF4α is involved in the proliferation, apoptosis, invasion, and migration of cancer cells both in vitro and in vivo [24–27]. Therefore, we hypothesized that AMPK activation targets HNF4α and binds to BCL-2 to exert anti-apoptotic effects and reduce brain damage after cardiopulmonary resuscitation.

In this research, we explored the protective effect of AMPK targeted activation of HNF4α on hippocampal CA1 region after cardiopulmonary resuscitation in rats, and used the oxygen–glucose deprivation/reperfusion (OGD/R) cell model combined with ChIP-sequencing and Dual-luciferase assay to further clarify the possible mechanism of AMPK/HNF4α neuroprotection.

## Materials and Methods

### Animal Preparation

All experiments were conducted in accordance with the Regulations for the Administration of Affairs Concerning Experimental Animals (China) and were approved and supervised by the Institutional Animal Care and Use Committee of Sun Yat-sen University (Approval number: SYSU-IACUC-2021-000972). Adult male Wistar rats weighing 300–350 g were purchased from the Laboratory Animal Center of Southern Medical University. All rats were fed in an environment with sufficient food and water freely accessible, constant temperature (23 ± 2 °C), and appropriate humidity (45–55%) under a 12-h light/dark cycle (lights on at 8:00 A.M.). The animals were randomly divided into five groups: a sham group (n = 18), a CA group (n = 33), a CA + AICAR group (n = 35), a CA + Compound C group (n = 24), and a CA + AICAR + BI 6015 group (n = 22) (Fig. S1).

### Intracerebroventricular (ICV) Injection

Intracerebroventricular injection was performed according to the monograph of George Paxinos et al. [28]. In brief, the rats were anaesthetized and then fixed in a stereotaxic apparatus (RWD Life Science, China). According to the doses used in previous studies [29, 30], either 0.1 µM/10 μl AICAR (MedChemExpress, Cat. HY-13417A, USA), 1 ng/10 μl Compound C (MedChemExpress, Cat.HY-13418A, USA), and 0.1 µM/10 μl BI 6015 (MedChemExpress, Cat.HY-108469, USA) or 10 μl vehicle (double distilled water) was injected into the lateral ventricle area at predetermined coordinates 2 h prior to inducing cardiac attest (AP, 1.2 mm from bregma; lateral, 1.5 mm; and vertical, 4.0 mm). For post-arrest AICAR treatment, bilateral intracerebroventricular injection of AICAR (0.1 µM/10 μl) and Compound C (1 ng/10 μl) were given immediately after cardiac arrest. The microsyringes were left in the injection site for 5 min to facilitate diffusion of the drugs, and then the needle was removed, the drilled hole was sealed, and the scalp was debrided before suturing.

### Experimental Procedures

Animals were anaesthetized by intraperitoneal injection of 3% pentobarbital (45 mg/kg). Additional doses (10 mg/kg) were administered at intervals of 1 h or when appropriate to maintain anaesthesia. A 14-gauge cannula (Abbocath-T) was intubated through the trachea, and the small animal ventilator (RODENT VENTILATOR 683, Harvard Apparatus, Inc., USA) was connected. Three electrode needles were inserted into the rat’s upper and lower limbs to record the lead II electrocardiogram. A 26 G I.V. Catheter (CHS-26, China) punctured the arteria saphena, great saphenous vein and arterial tube to measure arterial blood pressure, and an intravenous tube was used for drug administration.

The rat cardiac arrest model was established by the asphyxia method. The rats were injected with rocuronium bromide (0.15 mg/kg) intravenously to eliminate spontaneous breathing, the ventilator was closed, and the trachea was blocked at the end of exhalation. When the mean arterial pressure (MAP) dropped to 30 mmHg (1 mmHg = 0.133 kPa), the asystole timer was started. A dose of epinephrine (0.02 mg/kg) was given 8 min later. At the same time, the airway was opened, and mechanical ventilation was established with pure oxygen at a tidal volume of 6 mL/kg at a frequency of 80 times/min. Chest compressions were immediately performed at a frequency of 200 times/min, and the compression depth was 1/3 of the anteroposterior diameter of the thorax. If no return of spontaneous circulation (ROSC) was observed after 3 min, another dose of epinephrine (0.02 mg/kg) was added intravenously. Resuscitation was stopped when ROSC failed after 30 min. Return of spontaneous circulation was defined as a supraventricular rhythm with MAP more than 90 mmHg that lasted for at least 30 min.


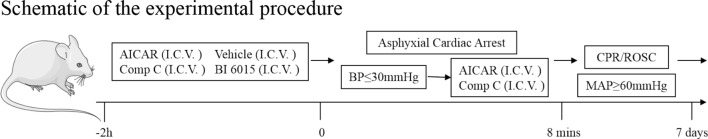


### Primary Cortical Rat Neuron Culture

Primary cortical neurons were prepared from 1-day-old Wistar rat pups. Briefly, the whole brain was removed aseptically, and a piece of cortical tissue was dissected out. Then, the blood vessels and meninges were removed. After cutting up the cortical tissues with sterile scissors, cerebral cortices were digested in 0.25% trypsin–EDTA (GIBCO, Cat. 25200072, USA) for 10 min, blown with a Pasteur pipette, and then filtered with a 70-micron filter. The cell pellet obtained by centrifugation was resuspended in DMEM containing 10% foetal bovine serum and plated at a density of 1 × 10^7^ cells/mL on poly-l-lysine-coated 6-well (2 mL/well) plates and 24-well (0.5 mL/well) plates. After plating for 24 h, unattached cells and debris were removed by replacing the initial medium with fresh neurobasal medium (GIBCO, Cat. 10888022, USA) containing B-27 supplements (GIBCO, Cat. 17504044, USA), glutamine (0.5 mM, Maclin G6203, China), and cytosine arabinoside (5 μM, Selleck, Cat.S1648, China). Twenty-four hours later, the initial neurobasal medium was replaced. The primary cortical neurons were cultured in an incubator at 37 °C and 5% CO_2_ humidity, and half of the medium was replaced every 2 days. The follow-up experiment was performed after 7 days of culture (the cell morphology is shown in Fig. S2B).

### PC12 Cell Culture

The PC12 cells were purchased from iCell Bioscience, Inc., Shanghai, and were cultured in Dulbecco’s Modified Eagle’s Medium (DMEM, Gibco, Cat. C11995500BT, USA) supplemented with 10% foetal bovine serum (ExCell Bio, Cat.FSP500, China) and 1% penicillin‒streptomycin (Gibco, Cat. 15140122, USA). As described in a previous experiment [31], PC12 differentiation was induced using nerve growth factor (NGF, Gibco, Cat. 13257019, USA) at a concentration of 100 ng/mL and cultured in a cell culture incubator at 37 °C with a humidity of 5% CO_2_/95% air for 3 days (the cell morphology is shown in Fig. S2A). To improve the reproducibility of the experiment, we used PC12 cells to explore the drug concentrations of AICAR, Compound C, benfluorex hydrochloride (BF, MedChemExpress, Cat. HY-B1058A, USA), and BI 6015 (Fig. S3A).

### Oxygen and Glucose Deprivation/Reperfusion (OGD/R)

Two cell types, primary cortical neurons and NGF-induced PC12 cells, were used in this experiment. Briefly, plates containing cells were washed twice with phosphate-buffered saline (PBS, pH 7.4) and then refreshed with glucose-free DMEM (Solarbio, Cat. 90113, China), transferred into a modular chamber (Fig. S4), and flushed with 5 L/min of a 95% N_2_ and 5% CO_2_ gas mixture for 10 min at room temperature. Next, the chamber was sealed and placed in a 37 °C incubator for OGD experiments. According to our previous time exploration (Fig. S3C, D), primary cultures of cortical neurons underwent OGD for 2 h/PC12 cells underwent OGD for 4 h and were then changed to normal medium. Primary cultures of cortical neurons were reperfused for 24 h/PC12 cells were reperfused for 6 h. Cells without any treatment served as the control group.

### Dataset Collection and Analysis

Two datasets, GSE50815 and GSE53862, were obtained from the GEO dataset using “chip-seq” and “HNF4α” as keywords, of which GSE50815 contains the sequencing information of one kidney tissue sample (SRR980344), and GSE53862 contains the raw data of two hepatocyte samples (SRR1103818, SRR1103819). The sequencing data were processed through the Computational pipeline (Fig. S5), and then the results were analysed by Gene Ontology (GO) and Kyoto Encyclopedia of Genes and Genomes (KEGG) pathway enrichment analysis (Fig. S6A–L). GO term analysis was classified into three subgroups, namely, biological process (BP), cellular component (CC) and molecular function (MF). Integrative Genome Viewer (IGV) software was used to visualize the genes of interest (Fig. S6N).

### Evaluation of Neurological Function

Neurological deficit scores (NDS) were assessed by two investigators unaware of the experimental groups at 24, 48, and 72 h after ROSC. NDS was assessed on a scale of 0–80 based on arousal level, cranial nerve reflexes, muscular tension, motor function, seizure, and simple behavioural responses. Normal behaviour was characterized by 80 points, while brain damage was characterized by 0 points. In this study, dead rats were excluded from the NDS assessment.

### Morris Water Maze Test

This test was used to evaluate the animal learning and memory abilities. In the training phase, each rat had three sessions of trials per day for 4 days in which they had to find a hidden platform (diameter 12 cm) placed in the centre of one of the quadrants of a tank and submerged 2 cm under the surface of the water. The latency to find the platform was measured in each trial. Between successive trials, there was a 10-min interval. All trials lasted no more than 60 s. If the rats had not discovered the platform in 60 s, they were manually guided to the platform. The CPR model was performed on Day 5, and 1 week after ROSC, all of the rats were subjected to a probe trial in which the platform was removed. The animals’ swimming path was measured for the quantification of latency, number of target crossings, time in the target quadrant, and swimming speed by the video tracking system TopScan (CleverSys Inc., Reston, VA, USA). The behavioural tests were performed by experimenters who were blinded to the experimental groups.

### Haematoxylin–Eosin (HE) and Nissl Staining

Twenty-four hours after CPR, the rats were anaesthetized with 3% sodium pentobarbital and then perfused with 4% paraformaldehyde via the apex. The brain tissue was fixed with paraformaldehyde for 24 h, dehydrated and embedded in paraffin, after which a 4-mm coronal section of the brain tissue was prepared. The sections were stained with haematoxylin–eosin or 1% toluidine blue (Nissl). Morphological and pathological changes in the brain tissue were observed under a microscope (CKX41, Olympus, Japan), and the number of intact neurons was counted.

### TUNEL Assay

TUNEL staining was performed using an In Situ Cell Death Detection Kit (Roche, Cat. 11684817910, Germany) according to the manufacturer’s instructions. Apoptotic cells were detected by fluorescence microscopy (DMi8, Leica, Germany). TUNEL-positive neurons/DAPI were regarded as an apoptosis index.

### CCK-8 Cell Viability Assay

A Cell Counting Kit (MedChemExpress, Cat. HY-K0301, USA) was used to assess cell survival. The experimental steps were strictly performed according to the manufacturer’s manual. Briefly, 50 μl of CCK-8 solution was added to 500 μl of medium solution in each neuronal culture well of a 24-well plate and incubated for 3 h at 37 °C. The absorbance at 450 nm was measured with a microplate reader (Sunrise, TECAN, Austria).

### Flow Cytometry

After OGD treatment, the apoptotic cells were double-stained with propidium iodide (PI) and an Annexin V-FITC apoptosis detection kit (BD Biosciences, Cat. 556547, USA) according to the manufacturer’s protocol. Apoptosis was detected by a CytoFLEX flow cytometer (Beckman Coulter, USA). In addition, flow cytometry was used to detect the effect of drugs on cell viability without OGD intervention (Fig. S3B).

### Live-Dead Cell Staining Assay

Cell viability was assessed by the Live-Dead Cell Staining Kit (APExBIO, K2081, USA) according to the manufacturer’s instructions. Calcein-AM turns to calcein after esterase digestion in a viable cell, which can emit strong green fluorescence. In contrast, propidium iodide (PI) can reach the nucleus by passing through disordered areas of dead cell membranes and becomes intercalated with the DNA double helix of the cell to emit red fluorescence. Fluorescence images were visualized under a fluorescence microscope (DMi8, Leica, Germany).

### Quantitative Real-Time PCR (qRT‒PCR)

Total RNA was extracted from rat hippocampi using TRIzol (Invitrogen, Cat. 15596108, USA) according to the manufacturer’s protocol. Reverse transcription was performed using the PrimeScript RT master mix reagent kit (TaKaRa, Cat. RR047A, Japan) to obtain cDNA. Next, qRT‒PCR was performed through the CFX96 Real-Time PCR System (Bio-Rad, USA) using TB Green® Premix Ex Taq II (Takara, Cat. RR820A, Japan). The fold change in mRNA expression for each gene was calculated using 2^−ΔΔC^. The primers used for amplification are presented in Table S1.

### ChIP-Seq Assay

Fresh cells (approximately 4 × 10^6^ cells per immunoprecipitation) were prepared, fixed with 1% formaldehyde for cell cross-linking and 125 mM glycine to terminate cross-linking, and harvested. Chromatin was sonicated, and 100–500-bp chromatin fragments were obtained. Chromatin fragments were incubated with 3.0 μg of HNF4α rabbit antibodies (Abcam, Cat, ab181604, USA) at 4 °C overnight to precipitate genomic regions bound to HNF4α. Protein A/G beads were added and incubated at 4 °C for 4–6 h, and Proteinase K was used to reverse the cross-links. Then, phenolic chloroform extraction and ethanol were used to purify DNA. Next, a ChIP sequencing library (Fig. S7) was constructed, and the purified products were sequenced using the Illumina protocol. Finally, the sequencing results were compared with the reference genome, and the sequence at the unique position on the alignment was used for subsequent standard information and personalized analyses (Fig. S8).

### Immunocytochemistry (ICC)

After 7 days of culture, the primary neuronal cells were detected by immunofluorescence. After washing with PBS, 4% paraformaldehyde solution was added and fixed at room temperature for 15 min. Then, the cells were permeabilized with 0.1% Triton for 1 h at room temperature. Then, mouse anti-MAP2 primary antibody (Abcam, Cat.ab300646, USA) and rabbit anti-HNF4α primary antibody (Abcam, Cat.ab201460, USA) were incubated overnight. The next day, goat anti-mouse fluorescent secondary antibody (Abbkine, Cat. A23410, China) and goat anti-rabbit fluorescent secondary antibody (Abbkine, Cat. A23220, China) were incubated for 1 h at room temperature. Anti-fluorescence quenching tablets with DAPI (Sigma-Aldrich, Cat. F6057, USA) were used to seal the slices, and a laser scanning confocal microscope was used for immunofluorescence analysis.

### Luciferase Assay

PC12 cells were plated at 1 × 10^5^ cells/well on 24-well plates, cultured for 24 h, and then cotransfected with pcDNA3.1-HNF4α plasmid and pGL3-Bcl-2-promoter-luciferase plasmid or negative controls by using Lipofectamine 3000 Reagent (Invitrogen, Cat. 11668-027, USA). The firefly and Renilla luciferase activities were continuously measured according to the instructions of the dual-luciferase reporter gene detection kit (Yeasen, Cat. 11402ES60, China) 48 h after transfection. Construction of reporter plasmids was performed by Da Hong Biosciences Inc. (Guangdong, China).

### Western Blot Analysis

Rat hippocampal samples and primary cultures of cortical neurons were lysed in RIPA buffer (Thermo Fisher, Cat. 89901, USA) with protease and phosphate inhibitors (Thermo Fisher, Cat. 78442, USA) and then centrifuged at 12,000 rpm for 20 min at 4 °C. The protein content was measured, and samples were resuspended in loading buffer and denatured at 95 °C for 15 min. Equal amounts of protein were separated using SDS/PAGE and transferred to PVDF membranes (Millipore, Cat. ISEQ00010, USA) by electroblotting. The membranes were blocked with 5% BSA for 2 h at room temperature, then cut horizontally with at least two markers on either side, and finally incubated overnight at 4 °C with primary antibodies. The following antibodies were used: AMPK (1:1000 dilution, Abcam, Cat. ab32047, USA), anti-AMPK (phospho S496) (1:1000, Abcam, Cat. ab92701, USA), HNF-4α (1:1000 dilution, Abcam, Cat, ab201460, USA), Bax (1:1000 dilution, Abcam, Cat. ab32503, USA), Bcl-2 (1:1000 dilution, Abcam, Cat. ab32370, USA), Cleaved-Caspase 3 (1:1000 dilution, CST, Cat. 9664T, USA), and β-actin (1:1000 dilution, Abcam, Cat. ab8227, USA). After 15 min with three washes with TBST (0.05% Tween 20), the membranes were incubated with goat anti-rabbit horseradish peroxidase (1:5000, Affinity, Cat. S0001, China) for 2 h at room temperature and then washed again. The protein signal was detected using Immobilon Western Chemiluminescent HRP Substrate (ECL) (Millipore, Cat. WBKLS0100, USA) reagent and visualized by an Amersham Imager 600 (GE, USA) and analysed using ImageJ software (NIH, Germany). If the two protein bands were very close, the membranes were stripped and reprobed. Put the strips into the TBST and washed the membranes on the shaker for three times, 5 min each time. The strips were placed in the Stripping Buffer (CWBIO, Cat. CW0056, China) and washed on a shaker for 30 min. The strips were transferred to TBST and washed on the shaker for three times, and then were re-blocked with 5% BSA. The following procedures were the same as before.

### Statistical Analysis

SPSS 25.0 (IL, USA) was applied for data analysis. Figures were drawn using GraphPad Prism 8 software. All the data are expressed as the mean value ± SD. Comparisons between the two groups used the *t* test. Comparisons among three groups or more were performed using one-way ANOVA. A *P* value < 0.05 was considered statistically significant.

## Results

### Basic Physiological Characteristics

The animal experiments in this study were divided into two parts. The first part included the sham group (n = 5), the CA + Vehicle group (n = 12), the CA + AICAR group (n = 12), and the CA + Compound C group (n = 12) (Table S2). The second part included the sham group (n = 5), the CA + Vehicle group (n = 12), the CA + AICAR group (n = 12), and the CA + AICAR + BI 6015 group (n = 12) (Table S3). There were no significant differences in body weight, heart rate, mean arterial pressure, body temperature, time from asphyxia to PEA and asystole, ventricular fibrillation times, cardiopulmonary resuscitation duration, or total epinephrine dosage among the experimental groups (Tables S2, S3).

### Activation of AMPK Improved Neurologic Recovery and Alleviated Neuronal Injury in the Hippocampal CA1 Region in Post-CA/CPR Rats

**Fig. 1:**
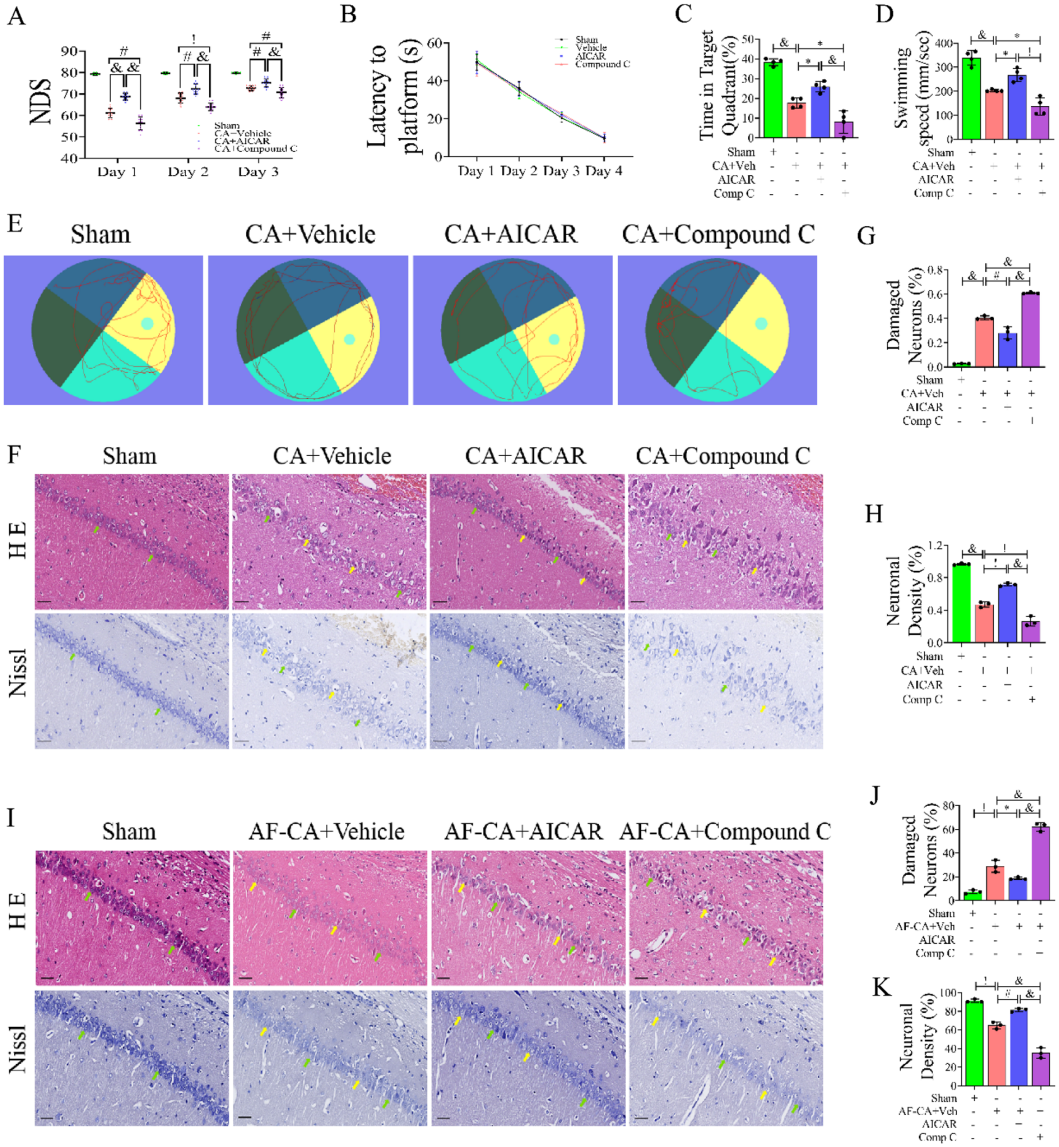
Effects of AMPK activation on neurological recovery and neuronal death in the hippocampal CA1 region in rats after CA/CPR. **A** Neurological deficit scores (NDS, 0 = brain death; 80 = normal) of surviving rats at 24, 48, and 72 h after ROSC. **B**–**E** The Morris water maze test was used to detect spatial learning and memory, n = 5. **B** In the training phase, the latency to find the underwater platform over 4 days was recorded. In the probe trial, the percentage of time in the target quadrant (**C**) and the average swimming speed of the rats (**D**) were recorded. **E** The swimming tracks of rats on the 8th experimental day. **F**–**K** HE staining (×200) and Nissl staining (×200) of hippocampal tissues CA1 region of rats from each group. The green arrows indicate surviving neurons, and the yellow arrows indicate damaged or dead neurons. Scale bar: 50 µm, n = 3, technical replicates = 2. Values are means ± SDs. & *P* < 0.0001, ^!^*P* < 0.001, ^#^*P* < 0.01, *P < 0.05. *AF-CA* + *Vehicle* injection vehicle after cardiac arrest, *AF-CA* + *AICAR* injection AICAR after cardiac arrest, *AF-CA* + *Compound C* injection compound C after cardiac arrest

Research has demonstrated that AMPK activation is not pronounced within 48 h after cardiopulmonary resuscitation (CPR), and AMPK activation has a protective effect on brain function after CPR [32]. We administered intraventricular injections of AICAR and compound C before cardiac arrest and measured neurological deficit scores for 3 consecutive days after the return of spontaneous circulation (ROSC). Compared with the CA + Vehicle group, the CA + AICAR group had higher NDS scores on Day 1 (*P* < 0. 0001), Day 2 (*P* < 0. 01), and Day 3 (*P* < 0. 05). The Morris water maze experiment was performed to assess the memory function of rats after cardiac arrest (Fig. 1B–E). Before cardiac arrest, the rats were trained for 4 days, and there was no significant difference in the latency time to reach the platform between each group (Fig. 1B). Seven days after CPR/ROSC, the water maze experiment was performed again, and the platform was removed. As shown in Fig. 1C, the CA + AICAR group stayed longer in the target area (*P* < 0.0001 versus CA + Compound C group). Figure 1D shows that the CA + AICAR group had a faster overall swimming speed (*P* < 0. 001 versus CA + Compound C group). Representative images (Fig. 1F) and statistical results (Fig. 1G) of HE staining showed that the neurons in the hippocampal CA1 region of the sham group were arranged in an orderly manner with normal structure, rich and uniform cytoplasm, and clear nucleoli. Many swollen neurons with loosened structures or vacuolar structures and karyopyknosis could be observed after CA. The pathological changes in hippocampal neurons were ameliorated in the CA + AICAR group, but hippocampal formation was further damaged in the CA + Compound C group, the arrangement of neurons was more disordered, and the morphology changed significantly. Representative images (Fig. 1F) and statistical results (Fig. 1H) of Nissl staining showed that the number of positive neuronal cells in the CA1 hippocampus decreased significantly after CA (*P* < 0. 01) and significantly increased after administration of AICAR (*P* < 0. 05), and the number of neurons was further reduced after switching to Compound C (*P* < 0. 05).

In addition, to further evaluate the role of AMPK activity on neuronal function after cardiac arrest, intracerebroventricular injection of AICAR was performed in rats after cardiac arrest. As shown in Fig. 1I–K, the pathological changes of hippocampal neurons were improved with AICAR treatment after cardiac arrest, and the number of abnormal neurons decreased. Injection of Compound C further damaged the neural structure of the hippocampus and reduced the number of normal neurons. Taken together, Activation of AMPK attenuated neuronal injury in the hippocampal CA1 region after cardiac arrest.

### HNF4α Played a Key Role in Brain Protection in the AMPK/HNF4α Pathway

**Fig. 2:**
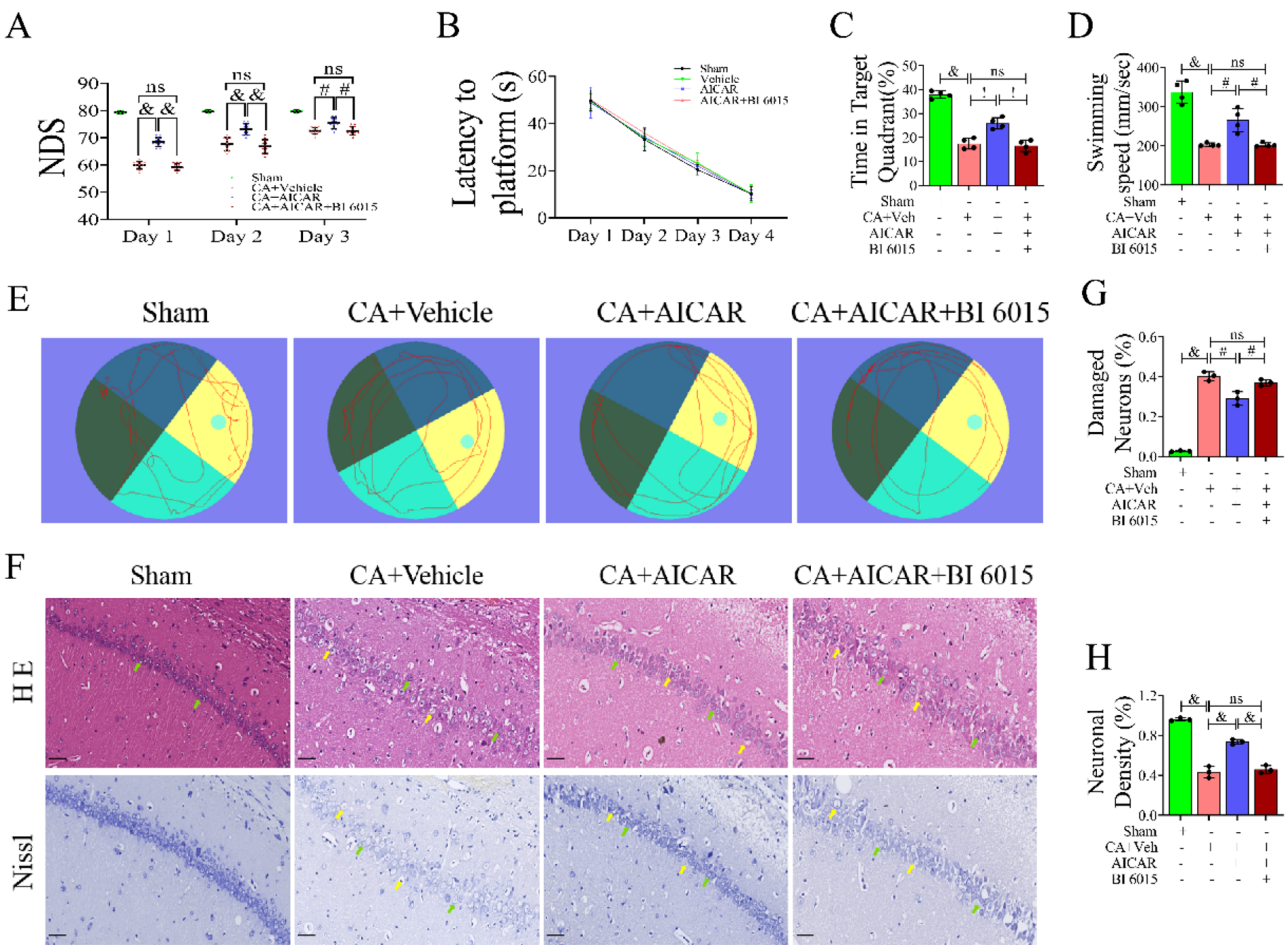
HNF4α inhibitor nullified the protective effect of AMPK on hippocampal CA1 neurons after cardiopulmonary resuscitation in rats. **A** Neurological deficit scores (NDS, 0 = brain death; 80 = normal) of surviving rats at 24, 48, and 72 h after ROSC. **B**–**F** The Morris water maze test was used to detect spatial learning and memory, n = 5. **B** In the training phase, the latency to finding the underwater platform over 4 days was recorded. In the probe trail, the number of times that the rats crossed the platform (**C**), the percentage of time in the target quadrant (**D**) and the average swimming speed of the rats (**E**) were recorded. (F) Swimming tracks of rats on the 8th experimental day. **G**, **H** HE staining (×200) and Nissl staining (×200) of hippocampal tissues CA1 regions of rats from each group. The green arrows indicate surviving neurons, and the yellow arrows indicate damaged or dead neurons. Scale bar: 50 µm, n = 3, technical replicates = 2. Values are means ± SDs. ^&^*P* < 0.0001, ^!^*P* < 0.001, ^#^*P* < 0.01, *P < 0.05, ^ns^*P* > 0.05

Recent emerging research has demonstrated that HNF4α may serve as a novel diagnostic and prognostic biomarker and an effective target for cancer therapy [33]. Li and Chen reported that the overexpression of HNF4α can promote the proliferation, migration and invasion of SH-SY5Y cells [27], and HNF4α can be phosphorylated by the upstream molecule AMPK to regulate transcriptional activity [34]. To clarify whether HNF4α plays a key role in transcriptional regulation, we found two datasets (GSE50815 and GSE53862) in NCBI GEO datasets, of which three samples, SRR980344, SRR1103818, and SRR1103819, contained HNF4α-related ChIP-seq sequencing results. We used a computational pipeline (Fig. S5) to perform GO enrichment analysis and KEGG pathway enrichment analysis on sequencing data (Fig. S6A–L). The enriched BP terms included forebrain development, proteasomal protein catabolic process, coenzyme metabolic process, regulation of protein serine/threonine kinase activity, and glucose homeostasis (Fig. S6A, E, I). Neuron-to-neuron synapse was the common enriched CC term (Fig. S6B, F, J). The main enriched MF terms were DNA-binding transcription activator activity, RNA polymerase II-specific activity, and transcription coregulator activity (Fig. S6C, G, K). KEGG pathway enrichment analysis showed that the AMPK signalling pathway was a common pathway in the three samples (Fig. S6D, H, L). According to Western blot analysis, AMPK was located upstream of HNF4α and had a positive correlation with HNF4α (Fig. S6M). We constructed the AMPK overexpression group and the HNF4α inhibition group by lateral ventricle injection of AICAR and BI6015 (an HNF4α inhibitor) and determined the three-day neurological function score (Fig. 2A) after CA/ROSC. Compared with the CA + Vehicle group, there was no significant difference in the three-day score of the CA + AICAR + BI6015 group (*P* > 0.05). Then, the Morris water maze experiment was performed to evaluate the memory function of the rats after cardiac arrest (Fig. 2B–E). Before cardiac arrest, rats were trained for 4 days, and the latency time of each group to reach the platform shown in Fig. 2B was not significantly different (*P* > 0.05). Seven days after ROSC, the water maze experiment was performed again, and the platform was removed. Compared with the CA + Vehicle group, the time spent in the target quadrant (Fig. 2C) and the total swimming speed (Fig. 2D) of the CA + AICAR + BI6015 group were not significantly different (*P* > 0.05). Representative images of HE staining (Fig. 2F) showed that compared with the sham group, the neurons in the hippocampal CA1 regions of the CA + Vehicle and CA + AICAR + BI6015 groups had loose or vacuolar structures and karyopyknosis. There was no significant difference in the proportion of neurons injured (Fig. 2G) (*P* > 0.05, CA + AICAR + BI6015 group versus CA + Vehicle group). According to the representative images of Nills staining (Fig. 2F) and the statistical results (Fig. 2H), compared with the CA + Vehicle group, the number of positive neurons in the hippocampal CA1 region of the CA + AICAR + BI6015 group showed no significant change (*P* > 0.05). Overall, the use of HNF4α inhibitors counteracted the protective effect of AMPK on hippocampal CA1 neurons after CPR.

### HNF4α Antagonist Attenuated the Protective Effect of AICAR Treatment by Increasing Apoptosis in Rats After CA/CPR

**Fig. 3:**
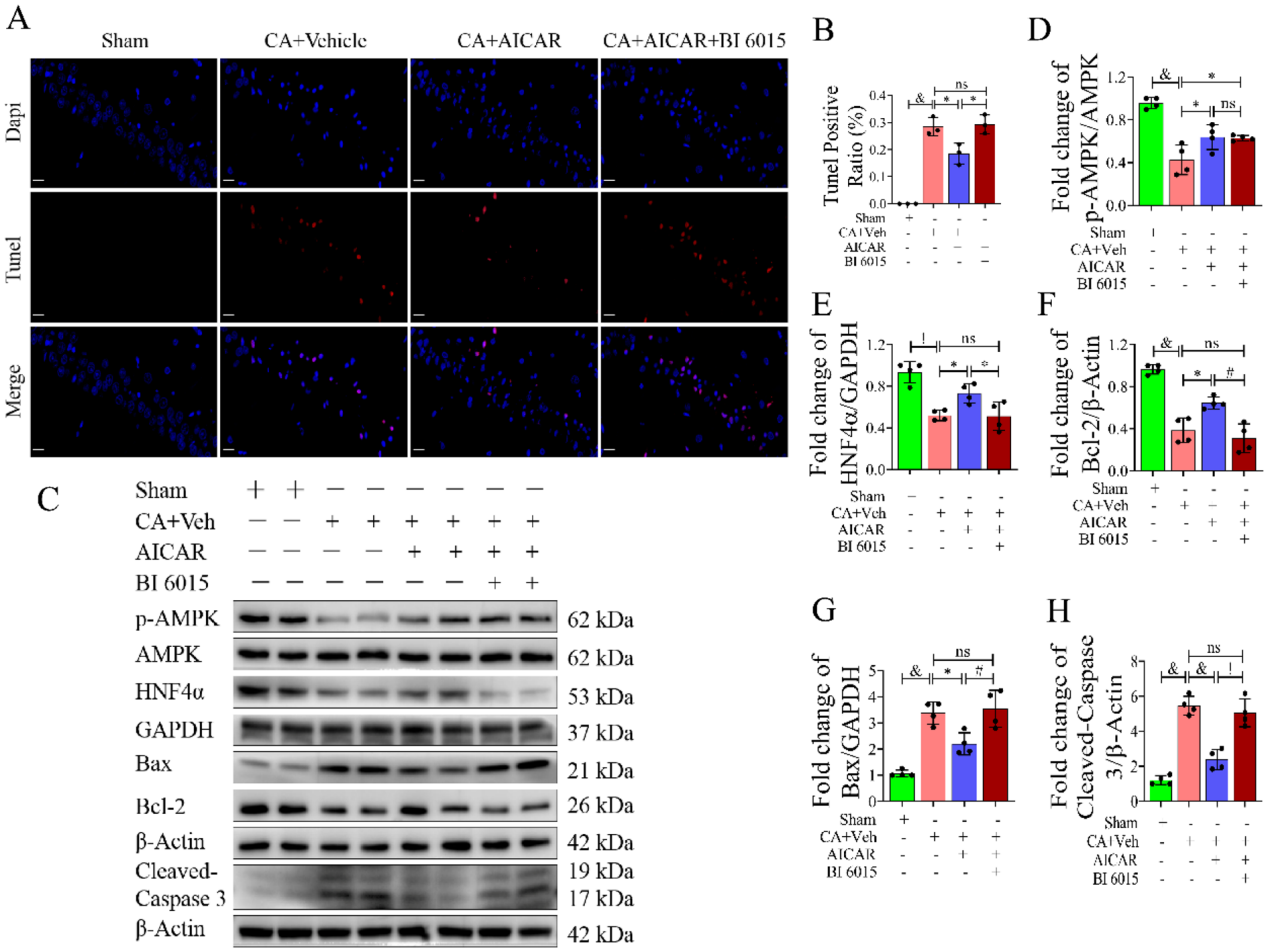
HNF4α affected AICAR-induced brain protection in rats after CPR through the apoptotic pathway. **A** TUNEL staining (×400) of hippocampal tissues in the CA1 regions of rats from each group. Positive TUNEL staining (red) is an indication of apoptosis. Scale bar: 20 µm. **B** Quantification of TUNEL-positive cells in the CA1 region of the hippocampus after CA/CPR in the rat model, n = 3, technical replicates = 2. **C**–**H** The expression levels of crucial apoptotic proteins in the rat hippocampus from each group were detected by Western blot, n = 4. Values are means ± SDs, ^&^*P* < 0.0001, ^!^*P* < 0.001, ^#^*P* < 0.01, *P < 0.05, ^ns^*P* > 0.05

Recently, Huo et al. found that HNF4α was significantly correlated with cell viability, apoptosis and oxidative stress in Parkinson’s disease [35]. We used IGV software to analyse the three SRR datasets previously described, showing that HNF4α may have a binding site with the antiapoptotic gene Bcl-2 (Fig. S6N). Then, we performed the TUNEL assay (Fig. 3A, B), which showed that the neurons in the hippocampal CA1 region underwent significant apoptosis after CA (*P* < 0.0001, versus sham group), and the number of apoptotic cells decreased after the use of AICAR (*P* < 0.05, versus CA group). On the basis of the use of AICAR, neuronal apoptosis was similar to that of the CA group when BI 6015 was added (*P* > 0.05). Next, we performed Western blot experiments (Fig. 3C–H). The results showed that the expression of p-AMPK (*P* < 0.0001, versus sham group) and HNF4α (*P* < 0.001, versus sham group) decreased synchronously after CA; at the same time, the expression of the anti-apoptotic factor Bcl-2 (*P* < 0.001, versus sham group) decreased, and the expression levels of the pro-apoptotic factors Bax (*P* < 0.0001, versus sham group) and Cleaved-Caspase 3 (*P* < 0.0001, versus sham group) increased. After the administration of AICAR, the expression of HNF4α (*P* < 0.05, versus CA group) increased with the increase in p-AMPK (*P* < 0.01, versus CA group) expression. At this time, the expression of Bcl-2 (*P* < 0.01, versus CA group) also increased, and the expression of Bax (*P* < 0.001, versus CA group) and Cleaved-Caspase 3 (*P* < 0.0001, versus CA group) decreased. Interestingly, p-AMPK expression was not affected after BI 6015 (*P* > 0.05, versus CA + AICAR group) administration, while HNF4α (*P* < 0.05, versus CA + AICAR group) and Bcl-2 (*P* < 0.01, versus CA + AICAR group) expression levels were decreased, but the expression levels of Bax (*P* < 0.0001, versus CA + AICAR group) and Cleaved-Caspase 3 (*P* < 0.001, versus CA + AICAR group) were increased. Compared with the CA group, the expression levels of HNF4α, Bcl-2, Bax and Cleaved-Caspase 3 in the CA + AICAR + BI6015 group were not significantly different (*P* > 0.05). Collectively, HNF4α inhibitors countered any AMPK-induced cerebroprotective effects by increasing apoptosis.

### Activation of HNF4α by AMPK Reduced OGD-Induced Neuronal Apoptosis

**Fig. 4:**
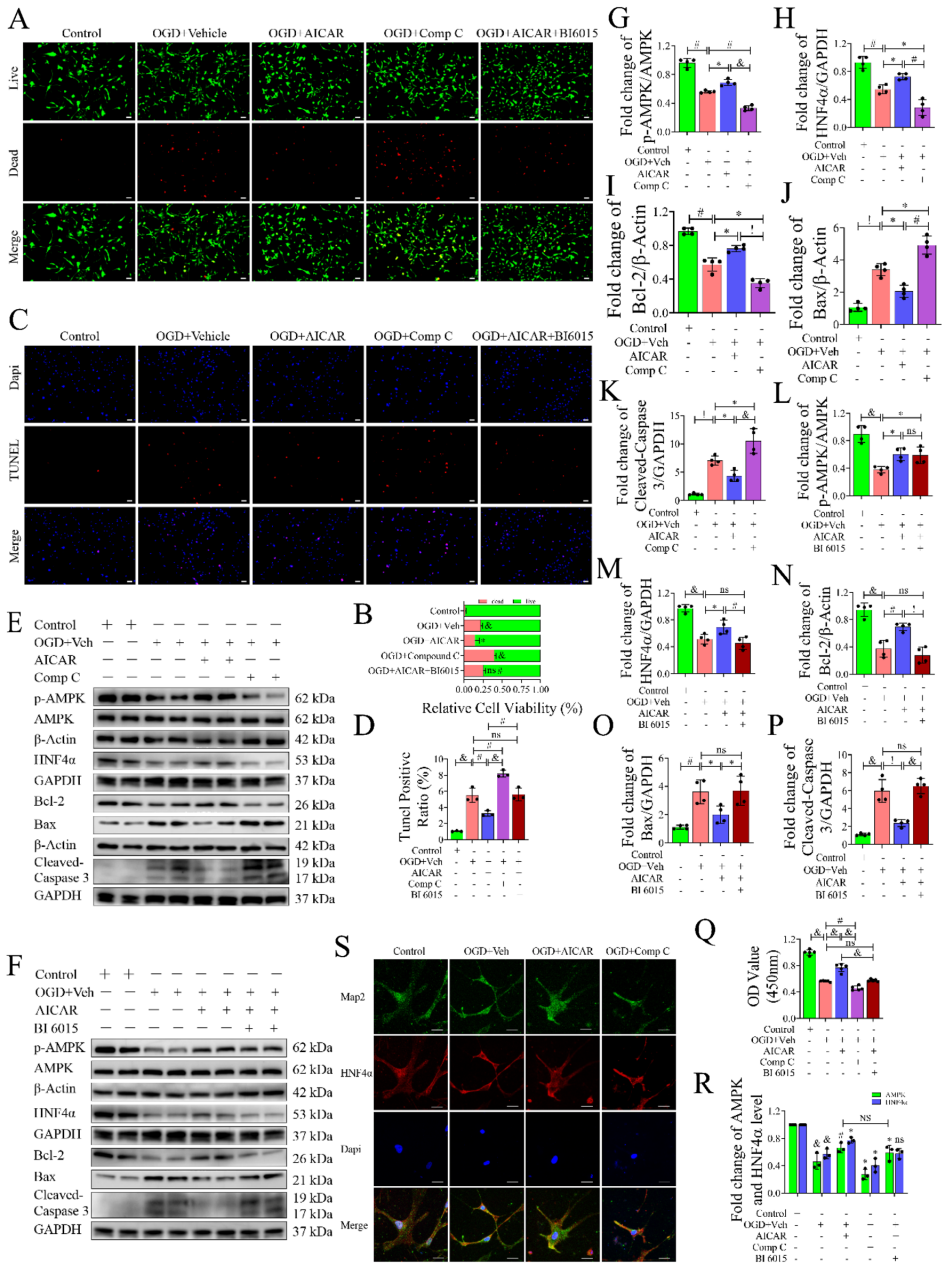
The role of AMPK/HNF4α pathway in OGD-induced neuronal apoptosis. **A** Live/dead cell staining assay (×100) to detect cell viability. Live cells were stained green, and dead cells were stained red. Scale bar: 200 µm. **B** Quantitative data of the live/dead staining assay, n = 3, technical replicates = 3. **C** Representative TUNEL staining (×100) image of neuronal apoptosis. Positive TUNEL staining (red) was an indication of apoptosis, and blue fluorescence represented the nucleus. Scale bar: 200 µm. **D** Quantification of apoptotic cells, n = 3, technical replicates = 3. **E**, **G**–**K** Identification of the effect of AMPK on neuronal apoptosis by Western blot, n = 4. **F**, **L**–**P** The effect of an HNF4α inhibitor (BI 6015) on neuronal apoptosis was detected by Western blot, n = 4. **Q** Cell viability was measured by a CCK8 kit (450 nm absorbance), n = 5. **R** The trend changes of AMPK and HNF4α mRNA levels were detected by qPCR using AICAR, Compound C, and BI 6015, respectively. n = 3, technical replicates = 3. **S** The fluorescence expression of HNF4α in neurons was detected by confocal microscopy. Values are means ± SDs, ^&^*P* < 0.0001, ^!^*P* < 0.001, ^#^*P* < 0.01, *P < 0.05, ^ns^*P* > 0.05

We further investigate the neuroprotective mechanism of AMPK/HNF4α in vitro. First, the optimal drug concentrations were explored in PC12 cells, which were AICAR: 100 µmol/L/12 h, Compound C: 1 µmol/L/12 h, BF (an HNF4α agonist): 0.02 nmol/L/24 h, and BI 6015: 5 µmol/L/24 h (Fig. S3A). Next, flow cytometry and electron microscopy were performed on primary cultures of cortical neurons to verify cell viability using the drug concentrations explored (Fig. S3B. S9), and the results showed that the above drug concentrations had no significant effect on normal cell viability. Then, the OGD/R time of primary neuronal cells was determined by CCK8 assay (Fig. S3C). Live/dead cell staining (Fig. 4A, B) showed that the number of dead cells decreased after treatment with AICAR (*P* < 0.05, versus OGD group), while the number of dead cells increased significantly after Compound C administration (*P* < 0.0001, versus OGD group). Moreover, the protective effect of AICAR on cells was partially inhibited by BI 6015 (*P* < 0.01, versus OGD + AICAR group). Similarly, in the TUNEL staining experiment (Fig. 4C, D), neuronal apoptosis was decreased after AICAR administration (*P* < 0.01, versus OGD group) and increased after Compound C treatment (*P* < 0.01, versus OGD group). Compared with the OGD + AICAR group, the apoptosis of neurons was also significantly increased after adding BI 6015 (*P* < 0.01). Next, Western blot analysis was performed to investigate the expression of AMPK, HNF4α and related apoptotic genes (Fig. 4E, F). The expression of HNF4α increased with the phosphorylation of AMPK, and the increase in HNF4α promoted the expression of Bcl-2 and inhibited the expression of Bax and Cleaved-Caspase 3 (Fig. 4G–K). Interestingly, the expression of AMPK was not significantly changed with an HNF4α inhibitor (BI 6015) (*P* > 0.05, versus OGD + AICAR group) (Fig. 4L), while the expression levels of HNF4α (*P* < 0.01, versus OGD + AICAR group) and Bcl-2 (*P* < 0.0001, versus OGD + AICAR group) were significantly decreased (Fig. 4M, N) and the expression levels of Bax (*P* < 0.05, versus OGD + AICAR group) and Cleaved-Caspase3 (*P* < 0.0001, versus OGD + AICAR group) increased (Fig. 4O, P). Subsequently, we performed a CCK8 assay (Fig. 4Q, R) using the same grouping and found similar results to the Live/Dead cell staining assay. The AICAR treatment group had better cell viability after OGD/R than the Compound C group (*P* < 0.0001), while the viability of cells was decreased after BI 6015 treatment (*P* < 0.01, versus OGD + AICAR group). Furthermore, qPCR analysis showed that AMPK positively regulated the expression of HNF4α, and the use of an HNF4α inhibitor (BI 6015) had little effect on AMPK (Fig. 4S). Using immunofluorescence confocal microscope analysis, we found that the expression of HNF4α in the cytoplasm and nucleus decreased after OGD. Nuclear translocation of HNF4α occurred after AICAR administration, and the expression of HNF4α in the nucleus was significantly increased compared with that in the OGD group. However, the expression of HNF4α in the nucleus was reversed after the use of an AMPK inhibitor (Compound C) (Fig. 4T). In general, activation of HNF4α by AMPK increased cell activity and reduced neuronal apoptosis.

### HNF4α Bound to Bcl-2 Inhibited OGD-Induced Neuronal Apoptosis

**Fig. 5:**
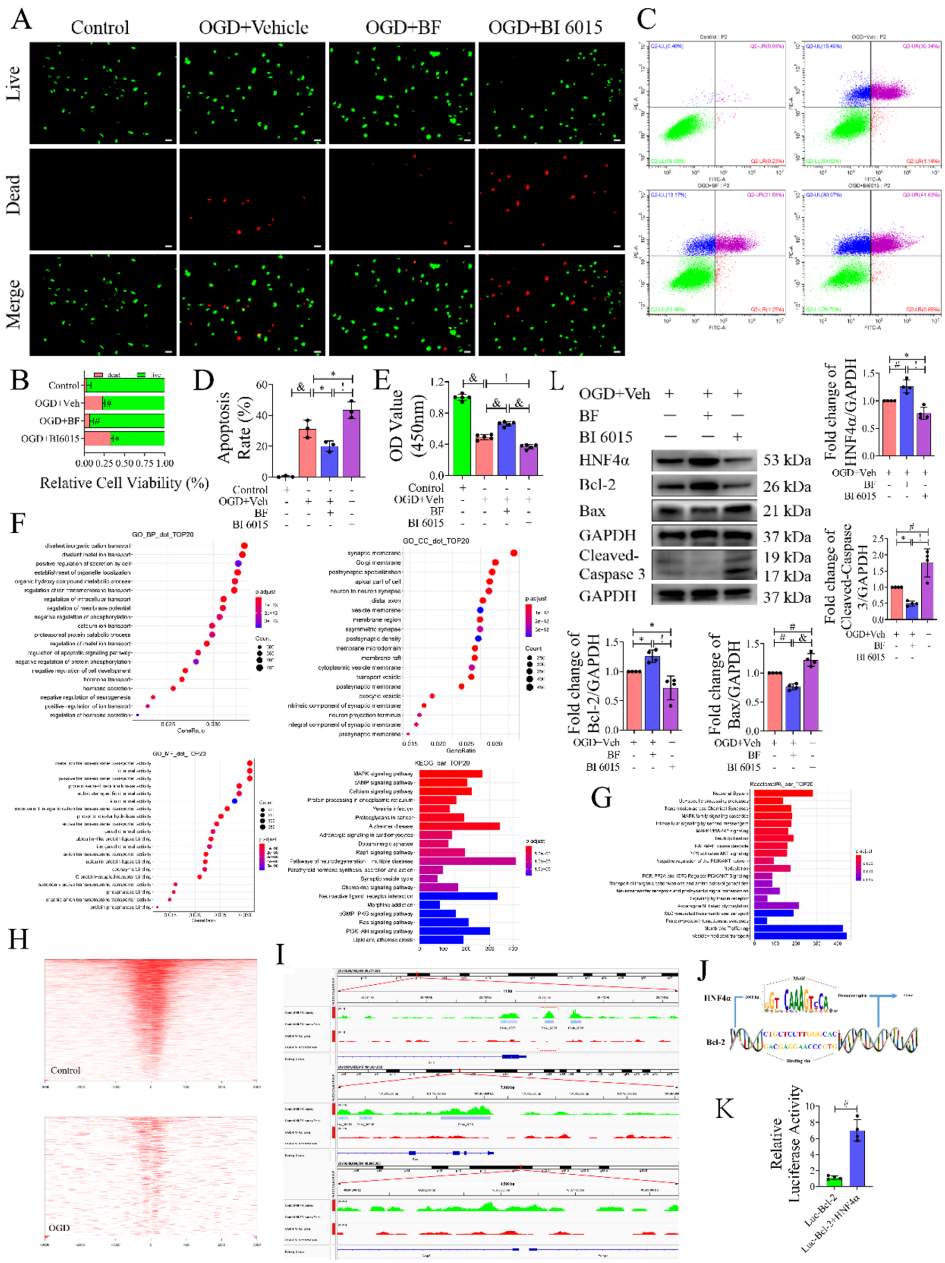
HNF4α combined with BCL-2 exerts an anti-apoptotic effect. **A** Live/dead cell staining assay (×100) to detect cell viability. Live cells were stained green, and dead cells were stained red. Scale bar: 200 µm. **B** Quantitative data of the live/dead staining assay, n = 3, technical replicates = 3. **C** Representative images of apoptosis were detected by flow cytometry after neuronal OGD/R. As shown in the figure, the lower left quadrant (Q2-LL) represents normal living cells, the upper left quadrant (Q2-UL) represents cell debris or dead cells due to other reasons, the upper right quadrant (Q2-UR) represents late apoptotic neurons, and the lower right quadrant (Q2-LR) represents early apoptotic neurons. **D** The percentage of neuronal apoptosis after OGD/R was calculated by flow cytometry, n = 3, technical replicates = 2. **E** Cell viability was measured by a CCK8 kit (450 nm absorbance), n = 5. **F** The top 20 GO terms and the top 20 KEGG pathway terms were significantly enriched in the ChIP-seq analysis of well-differentiated PC12 cells. GO term enrichment analysis included biological process (BP), cellular component (CC), and molecular function (MF) terms. **G** Top 20 Reactome pathway analyses. **H** Heatmap distribution of peak enrichment intensity at the proximal TSS. **I** The genomic regions around the Bcl-2, Bax and Caspase 3 promoters were visualized through the Integrative Genome Viewer (IGV). **J** Schematic diagram of HNF4α motif binding to the Bcl-2 promoter sequence. **K** A dual-luciferase reporter assay was conducted to investigate the targeting relationship between HNF4α and Bcl-2 in well-differentiated PC12 cells. n = 4, technical replicates = 6. **L** The expression of crucial apoptotic proteins after OGD/R was detected by Western blotting and statistical analysis, n = 4. Values are means ± SDs, ^&^*P* < 0.0001, ^!^*P* < 0.001, ^#^*P* < 0.01, *P < 0.05, ^ns^*P* > 0.05

We further verified the effect of HNF4α on neurons after OGD using an HNF4α agonist (BF, Benfluorex Hydrochloride) and an inhibitor (BI 6015). In live/dead cell staining experiments (Fig. 5A, C) and CCK8 assays (Fig. 5E), we found that the use of BF significantly attenuated OGD-induced neuronal damage (P < 0.01, versus OGD group) and increased neuronal viability (P < 0.001, versus OGD group), while the use of BI 6015 aggravated neuronal damage (P < 0.05, versus OGD group) and inhibited neuronal viability (P < 0.01, versus OGD group). Next, PI and Annexin V double staining was used to detect the proportion of neuronal apoptosis by flow cytometry (Fig. 5B, D). The BF treatment alleviated OGD-induced neuronal apoptosis (P < 0.05, versus OGD group). However, the BI 6015 treatment group had significantly increased neuronal apoptosis (P < 0.001, versus OGD group). Furthermore, we performed ChIP-seq experiments using NGF-induced well-differentiated PC12 cells, followed by GO and KEGG pathway enrichment analyses (Fig. 5F). BP term enrichment analysis included regulation of intracellular transport and regulation of apoptosis signalling pathway. Synaptic membrane, postsynaptic specialization, and neuron-to-neuron synapse were the main CC term enrichment functions. MF term enrichment functions mainly included channel activity, and passive transmembrane transporter activity. ReactomePA enrichment analysis mainly focused on the neuronal system (Fig. 5G). The heatmap distribution of peak enrichment intensity at the proximal transcription start site (TSS) showed that the HNF4α binding site was significantly enriched on the TSS, and the enrichment intensity of the HNF4α binding site on the TSS was significantly reduced after OGD treatment (Fig. 5H). Next, using IGV software to analyse the ChIP-seq data (Fig. 5I), it was found that there are two binding sites in the upstream promoters of HNF4α, Bcl-2, Peak-6225 and Peak-3866, respectively. Combined with JASPAR analysis, Peak 6225 was more likely to be the binding site of HNF4α, with an adjusted P value of 13.28982 (calculated as − log10Pvalue, see Supplementary narrowpeak materials), and there were no HNF4α binding sites in the upstream promoters of Bax and Caspase 3. Figure 5J shows a schematic diagram of the binding of HNF4α to the Bcl-2 upstream promoter sequence. Subsequently, we performed a luciferase reporter gene assay driven by the Bcl-2 promoter, which demonstrated that HNF4α enhanced the expression of the Bcl-2 gene (Fig. 5K). Finally, Western blot analysis showed that HNF4α could promote the expression of Bcl-2, which negatively regulated the expression of Bax and Cleaved-Caspase 3 (Fig. 5L). In conclusion, HNF4α can positively regulate Bcl-2 to exert anti-apoptotic effects.

## Discussion

**Fig. 6:**
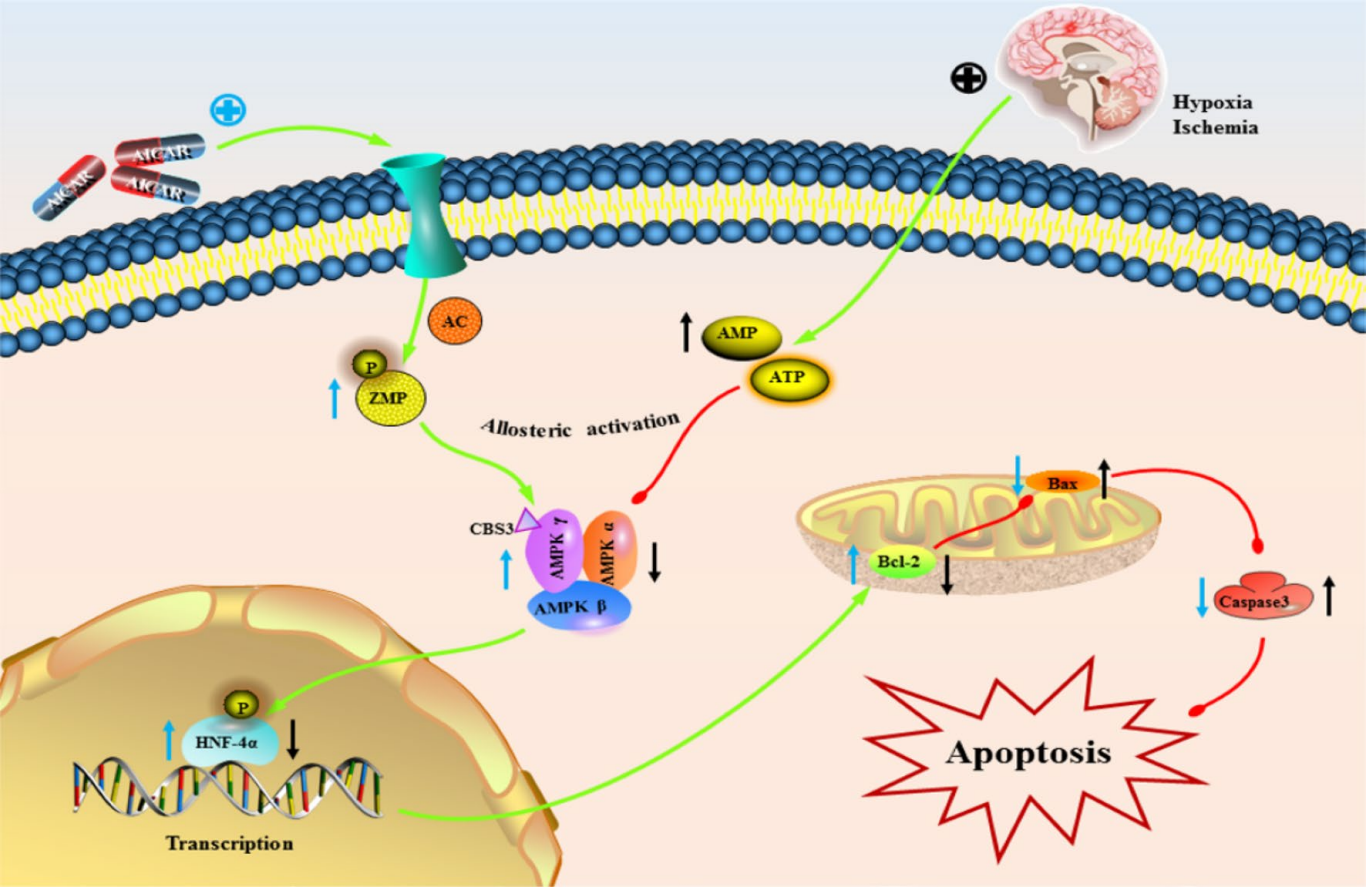
Schematic representation of the AMPK/HNF4α/Bcl-2 signalling pathway. In the early stage of ischaemia and hypoxia, the ratio of AMP/ATP is increased, and the activity of AMPK is inhibited, thereby downregulating the expression of HNF4α, which in turn affects the expression of Bcl-2 and aggravates cell apoptosis. Activation of AMPK after the use of AICAR further phosphorylates HNF4α, promotes the binding of HNF4α to the Bcl-2 promoter, increases the expression of Bcl-2, and inhibits the expression of Bax and Caspase 3, thereby reducing apoptosis

Our results showed that activation of AMPK attenuated brain injury and reduced neuronal apoptosis after CA and significantly improved three-day survival and seven-day spatial memory function in rats after ROSC. The reduction in AMPK and HNF4α might contribute to aggravated brain injury and increased apoptosis in rats after CA/CPR, and HNF4α plays a key role in mitigating neuronal apoptosis. Furthermore, AMPK activated the downstream gene HNF4α, thereby targeting Bcl-2 to attenuate apoptosis and neuronal injury in the hippocampal CA1 region (Fig. 6).

The most common mechanism of brain injury after cardiac arrest and resuscitation is global cerebral ischaemia and hypoxia caused by systemic hypoperfusion, which in turn leads to disruption of cellular function and membrane and pump dysfunction, and the resulting energy deficit can increase the AMP/ATP ratio to activate AMPK in the brain. Zhu et al. [32] found that the phosphorylation level of AMPK in the hippocampus gradually increased during the return of spontaneous circulation after CPR for 6–48 h. Interestingly, He et al. [36] showed that AMPK phosphorylation levels in brain tissue gradually decreased during 3–12 h after cardiac arrest reperfusion. Our study also showed that AMPK phosphorylation in the hippocampus decreased 4 h after cardiopulmonary resuscitation, increased after AICAR administration, and further decreased after the use of Compound C, which may be due to differences in animal models and cardiac arrest induction methods. Whether AMPK is increased or decreased after CA seems to be related to the fluctuation of ATP levels in the brain after ischaemic injury [37], though the exact mechanism is still unclear. AICAR is an adenosine analogue taken up into cells by adenosine transporters and phosphorylated by adenosine kinase, thus generating the AMP-mimetic, AICAR monophosphate (ZMP) (Fig. 6). ZMP does not change the ADP:ATP ratio or alter oxygen uptake. Similar to AMP, ZMP binds to site 3 on the AMPKγ subunit to activate AMPK [38]. AICAR has been shown to decrease apoptosis in different cells associated with various disease conditions [39, 40] [41]. Recently, Wang et al. [42] demonstrated for the first time that AMPK regulates apoptosis-related molecules by targeting HNF4α in prostate cancer, and our study also demonstrated that activation of the AMPK-HNF4α pathway attenuates cardiopulmonary resuscitation (CPR)-induced neuronal apoptosis. Liu et al. [41] and Du et al. [43] found that activation of AMPK could improve cognitive dysfunction in animal experiments. The medium- and long-term observation of rats also found that after 7 days of spontaneous circulation recovery, the limb activity and foraging function of rats essentially returned to normal, and the CA + AICAR group showed better spatial memory function in the water maze experiment. Consistent with the pre-treatment results, AICAR treatment after cardiac arrest improved the pathological changes of hippocampal neurons and reduced the number of abnormal neurons in rats.

HNF4α is a transcription factor that belongs to the nuclear hormone receptor superfamily, which becomes a direct regulator of gene expression through interaction with gene transcriptional regulatory elements and is involved in tissue-specific cell differentiation and energy metabolism [44]. Mutations in this gene have been associated with monogenic autosomal dominant noninsulin-dependent diabetes mellitus type I, which stresses the important role of HNF4α in energy metabolism [45]. In a genome-wide transcriptional examination of the adult mouse brain following transient hypoxia, Xu et al. [23] found that more than half of 1241 hypoxia-regulated genes specific to the cerebellum have binding sites for HNF4α in their promoters. HNF4α appears to activate the transcription of a large number of cerebellar-specific hypoxia-responsive genes, but its role in the cerebral hypoxia response remains unclear. Research has shown that HNF4α acts as an oncoprotein that converges on genes encoding anti-apoptotic oncogenes and cytokines and may promote cancer development [46]. HNF4α also activates the expression of multiple genes encoding cell adhesion molecules, extracellular matrix components, cytoskeletal proteins, and factors involved in cell survival and proliferation control [33]. Analysis of clinical neuroblastoma tissue samples by Xiang et al. revealed that HNF4α promoted the invasion, metastasis and angiogenesis of neuroblastoma cells by targeting matrix metalloproteinase 14 [47]. Deng et al. reported that the long noncoding RNA small nucleolar RNA host gene 16 exerts oncogenic effects through the miR-542-3p/HNF4α axis via the RAS/RAF/MEK/ERK signalling pathway to induce neuroblastoma growth [48]. The above research suggested that HNF4α may play a key role in neural tissue. In this study, we injected rats with AICAR (AMPK agonist) and BI 6015 (HNF4α inhibitor) into the lateral ventricle. After the return of spontaneous circulation in cardiopulmonary resuscitation, we found that adding BI 6015 attenuated the protective effect of AMPK on neurological function compared with AICAR alone. Further, we constructed HNF4α adeno-associated virus, which overexpressed HNF4α, increased the expression of anti-apoptotic protein Bcl-2 and decreased the expression of pro-apoptotic protein Bax (Fig. S10). In cell experiments, primary cultures of cortical neurons were treated with an HNF4α agonist (Benfluorex Hydrochloride, BF) and an inhibitor of OGD. The results showed that the apoptosis of HNF4α agonists was significantly reduced and that the cell survival rate was higher. Subsequently, we performed ChIP-seq, dual-luciferase experiments and Western blotting to further confirm that HNF4α positively binds Bcl-2 to exert antiapoptotic effects and found the binding sites of HNF4α and Bcl-2.

It should be noted that the current study still has some limitations. First, in this research, the levels of the brain injury biomarkers NSE and S100β in peripheral blood were not monitored, and their correlations with HNF4α were not analysed. However, the results of neurological function monitoring in this study have shown that blocking HNF4α can weaken the protective effect of AMPK, which is sufficient to prove that HNF4α is involved in the protection of nerve injury after ROSC. Second, considering the clinical application prospects and pharmacological effects of drugs, this study only used drugs for intervention, which can better provide an experimental basis for the clinical use of these drugs. HNF4α may be a potential intervention target for brain injury after CPR. Finally, this study did not use HNF4α-specific transgenic mice to study the in vivo mechanism to verify its biosafety, but the protection of brain injury after ROSC has a strict time window and requires timely intervention. In future research, we will consider choosing larger animals for verification, such as beagle dogs and pigs, to provide an experimental basis for clinical trials.

## Conclusion

In this research, we demonstrate that AMPK activates HNF4α directly targeting Bcl-2 to exert an anti-apoptotic effects, thereby alleviating neuronal injury in the hippocampal CA1 region of rats, and clearly identified the binding site of HNF4α and Bcl-2, which provides a new therapeutic target for the treatment of brain injury caused by CA.

## Supplementary Information

Below is the link to the electronic supplementary material.

Supplementary file1 (PDF 5345 KB)

Supplementary file2 (DOCX 282 KB)

## Data Availability

The data that support the findings of this study are available from the corresponding author, Chunlin Hu, upon reasonable request.
