# Supplementary material for: Targeted Activation of HNF4α by AMPK Inhibits Apoptosis and Ameliorates Neurological Injury Caused by Cardiac Arrest in Rats
Source: Neurochem Res. 2023 Jun 20;48(10):3129–45. doi: 10.1007/s11064-023-03957-1 (PMC10471732; doi:10.1007/s11064-023-03957-1)
Supplement: Supplementary file 1 — Supplementary file1 (PDF 5345 KB) [file 11064_2023_3957_MOESM1_ESM.pdf]

Supplementary Data

Figure S1.

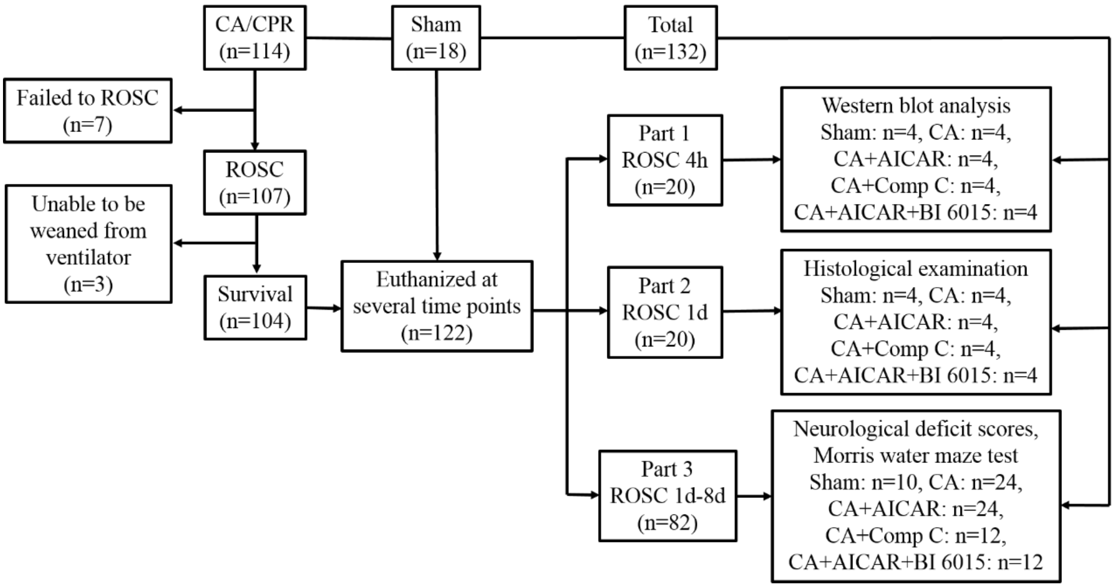

Figure S1. Flow chart of rat experiments.

A total of 132 rats were used in the experiment, of which 18 were in the sham group, and 114 were used to establish the CA/CPR model. After return of spontaneous circulation, 104 rats survived for subsequent experiments. The whole study consists of 3 parts. In part 1, Western blot was tested at 4 hour after ROSC. In part 2, histological examination (HE, Nissl, TUNEL assay) was analysed at day 1 after ROSC. In part 3, neurological deficit scores and morris water maze test were observed from day 1 to day 8 after ROSC.

Abbreviations: CA: cardiac arrest; CPR: cardiopulmonary resuscitation; ROSC: return of spontaneous circulation; AICAR: 5-aminidazole-4-carboxamide riboside, AMPK activator; Comp C: Compound C, AMPK antagonist; BI 6015: HNF4α antagonist.

Figure S2. Electron microscope comparison of NGF-induced PC12 cells and neurons

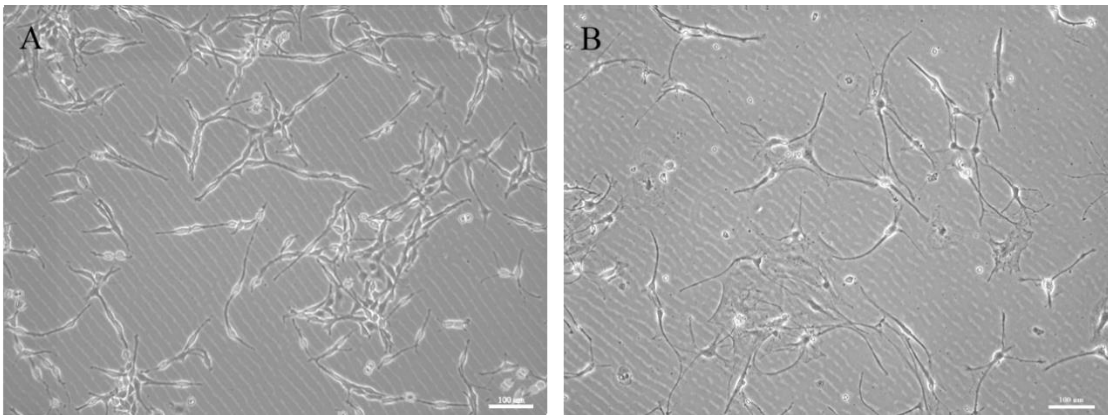

A: Morphology of NGF-induced PC12 cells, B: Morphology of neurons, scale bar: 100um

Figure S3. The optimal drug concentration and OGD/Reperfusion time were explored.

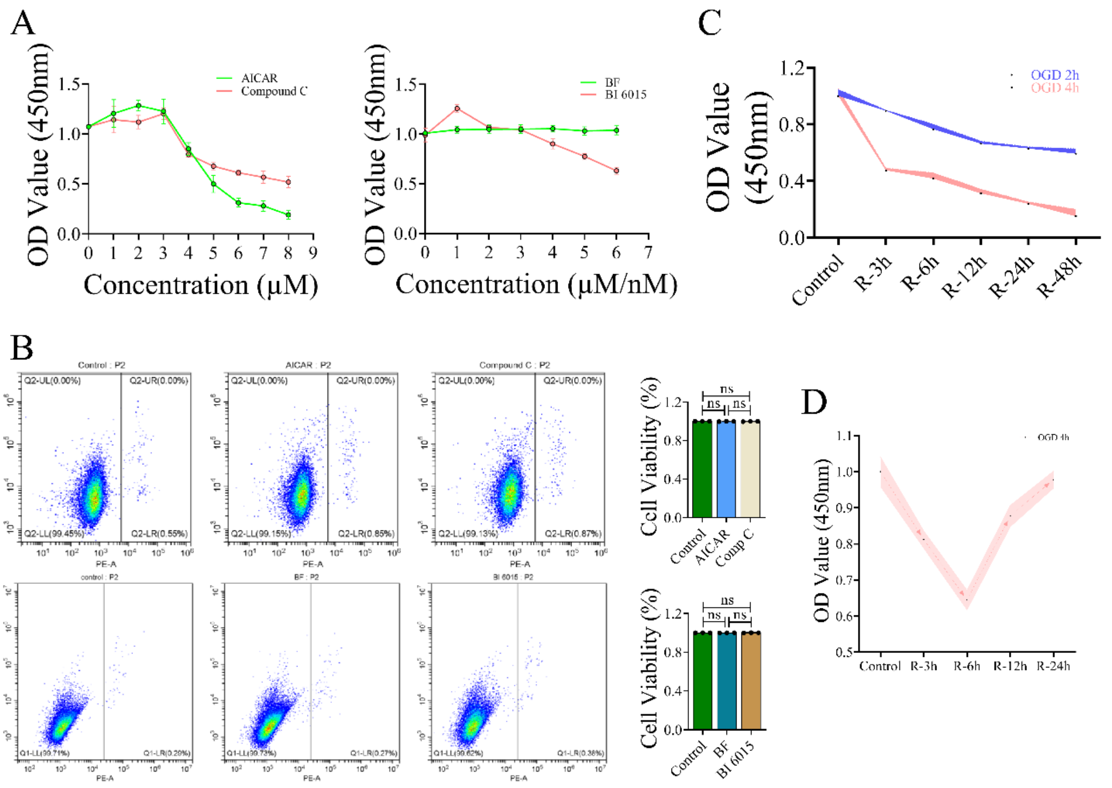

Figure S3. Exploring drug concentration and OGD/R time in cells.

(A) PC12 cells were used to explore the concentrations of AICAR, Compound C, BF and BI 6015 by CCK8 assay. The abscissa numbers 1 to 8 corresponded to the concentrations of AICAR and Compound C, respectively (AICAR: 20μmol/L, 50μmol/L, 100μmol/L, 200μmol/L, 400μmol/L, 600μmol/L, 800μmol/L, 1000μmol/L. Compound C: 0.2μmol/L, 0.5μmol/L, 1μmol/L, 2μmol/L, 4μmol/L, 6μmol/L, 8μmol/L, 10μmol/L). The abscess number 1 to 6 corresponded to the concentration of BF and BI 6015, respectively (BF: 0.01nmol/L, 0.02nmol/L, 0.05nmol/L, 0.1nmol/L, 0.2nmol/L, 0.25nmol/L. BI 6015: 1μmol/L, 5μmol/L, 10μmol/L, 15μmol/L, 20μmol/L, 25μmol/L). (B) flow cytometry was used to detect the viability of primary neuronal cells. (C) OGD/R time exploration of primary neurons by CCK8 assay. (D) OGD/R time exploration of NGF-induced PC12 cells by CCK8 assay. Values are means ± SD, n=3, ns $P > 0.05$.

Figure S4

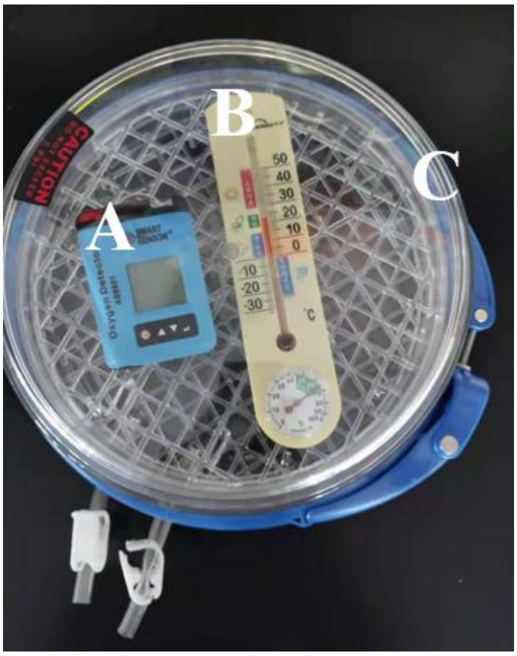

Figure S4. OGD experimental chamber system.

A. Oxygen meter, aerate the chamber at room temperature with a mixture of 95% N2 and 5% CO2 for 10 minutes. Close the valve when the oxygen meter shows oxygen concentration below 0.5%.

B. Thermometer, after the container is placed in the cell incubator, the temperature (37 ºC) inside is verified by a thermometer.

C. Modular Incubator Chamber (MIC-101, billups-rothenberg, USA).

Figure S5.

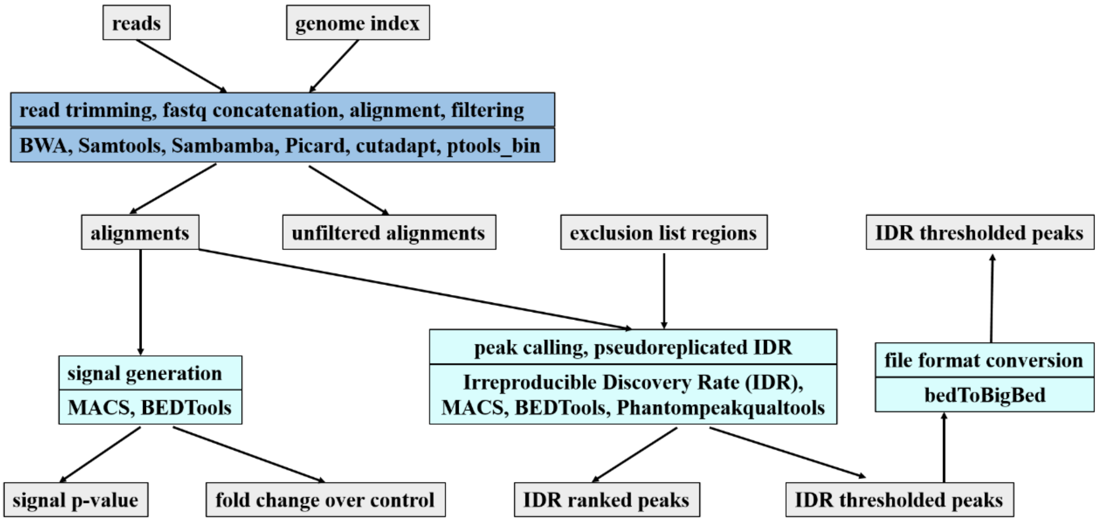

Figure S5. Computational pipeline schematic

Reference website: https://www.encodeproject.org/chip-seq/transcription-factor-encode4/

Figure S6. HNF4α may combine with Bcl-2 to participate in the regulation of AMPK signaling pathway in brain

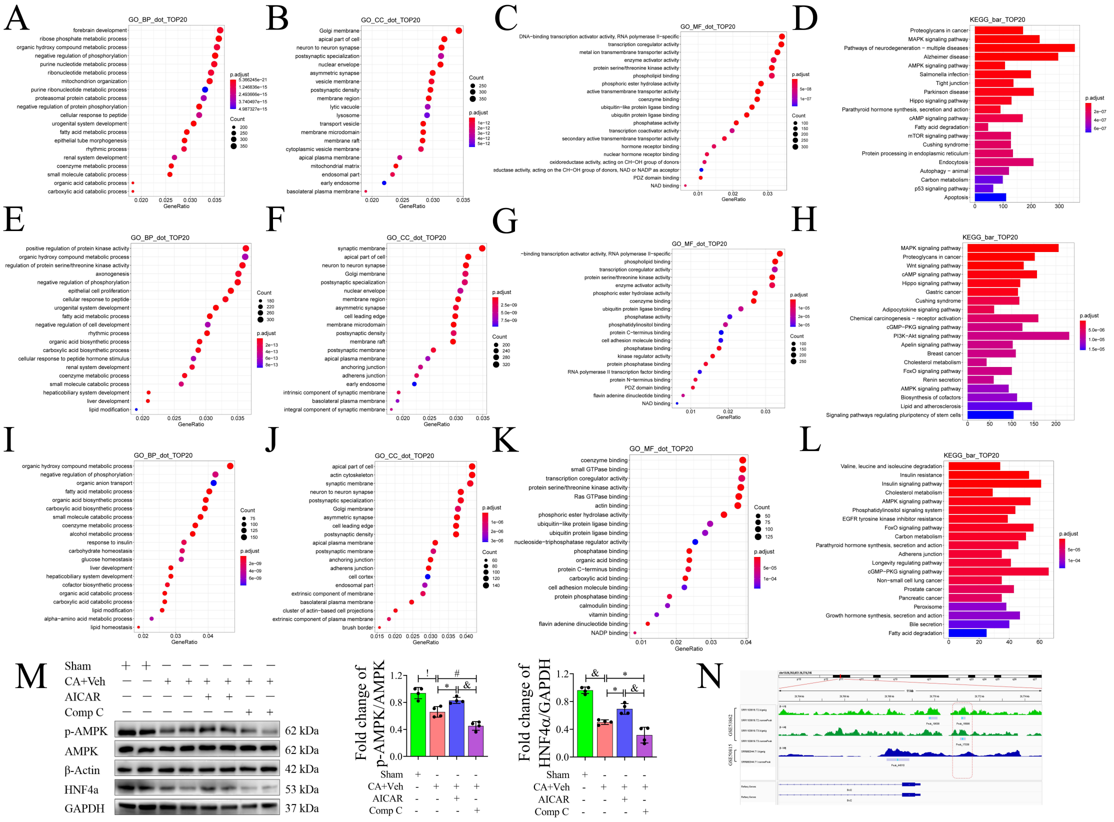

Figure S6. HNF4α played an important role in vivo and was activated by AMPK

Significantly enriched GO terms and KEGG pathway terms between the chip-seq analysis of kidney tissues and hepatocytes based on their functions. The top 20 GO terms and KEGG pathway terms in the enrichment analysis in three samples SRR980344 (A-D), SRR1103818 (E-H), SRR1103819 (I-L). (A, E, I) The top 20 biological process (BP) terms in the enrichment analysis, (B, F, J) The top 20 cellular component (CC) terms in the enrichment analysis, (C, G, K) The top 20 molecular function (MF) terms in the enrichment analysis. (D, H, L) The top 20 enriched Kyoto Encyclopedia of Genes and Genomes (KEGG) pathway terms of the three samples. (M) Representative western blot of AMPK and HNF4α expression in the hippocampus showed a positive correlation between AMPK and HNF4α, n=4. (N) The genomic region around Bcl-2 promoter was visualized through the Integrative Genome Viewer (IGV). Abbreviations: Gene Ontology (GO), Kyoto Encyclopedia of Genes and Genomes (KEGG) pathway. Values are means ± SD. & $P < 0.0001$, ! $P < 0.001$, # $P < 0.01$, * $P < 0.05$.

Figure S7

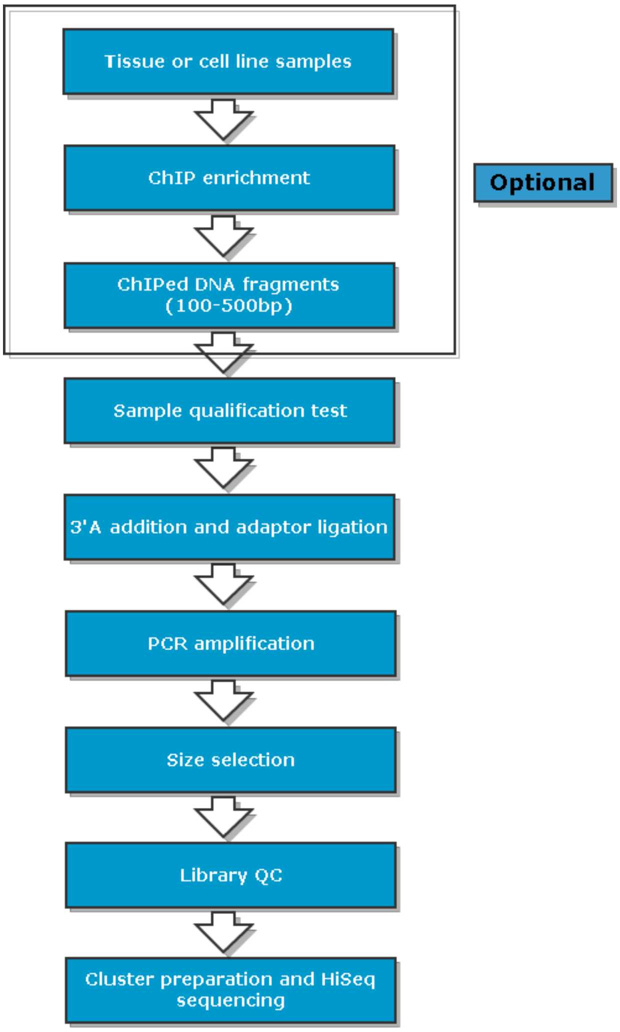

Figure S7. Chip-seq library construction process.

1. Quantitative detection of ChIP fragments with qubit.
2. Fill in the ends of the fragment and add an A tail to the 3' end.
3. Add Adapter.
4. 0.8X AMPure beads to remove excess Adapter.
5. Library PCR Amplification.
6. 1XAMPure beads to remove excess primer.
7. qPCR assay library concentration.
8. Agilent 2100 detects library fragment size.

Figure S8

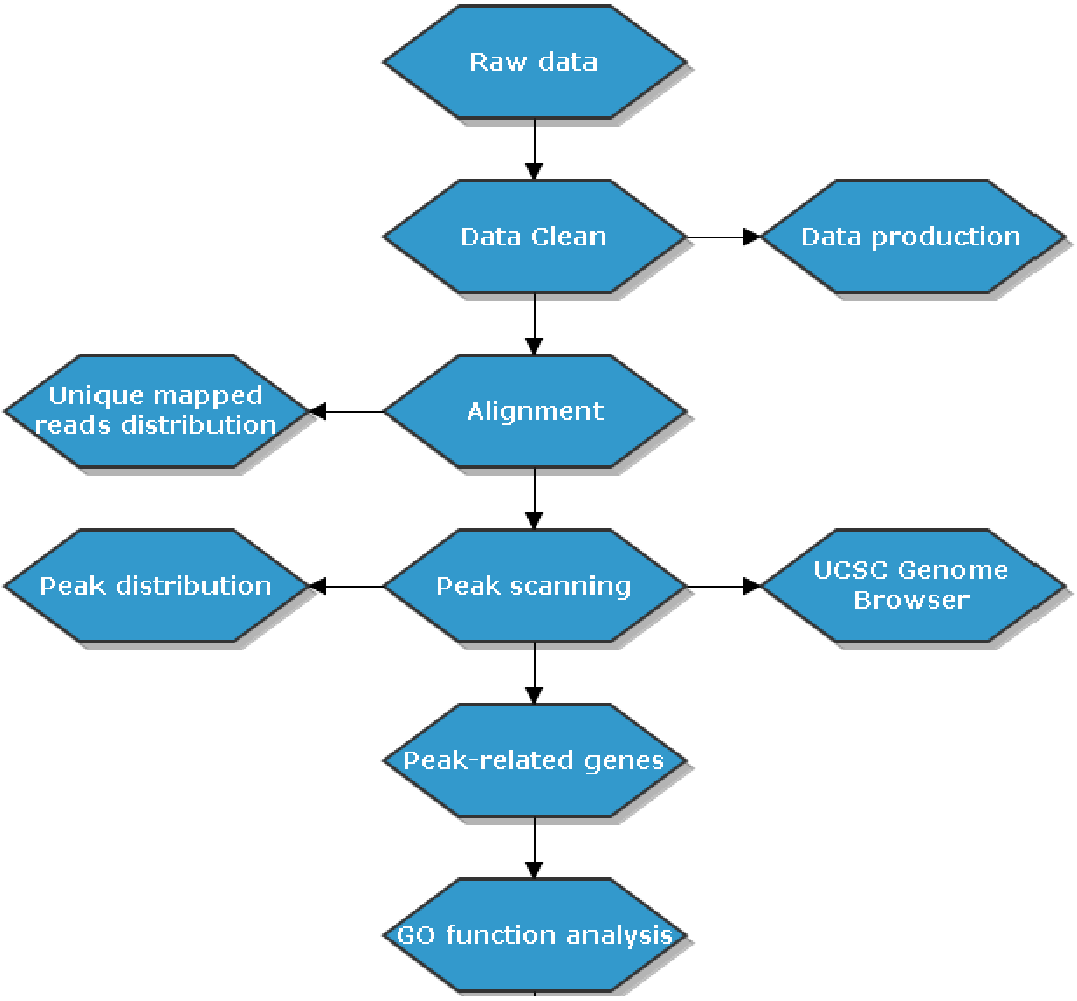

Figure S8. Bioinformatics Analysis Process

The sequencing results were compared with the reference genome, and the sequence at the unique position on the alignment was used for subsequent standard information analysis and personalized analysis.

Figure S9. The morphological changes of primary cortical neurons were observed by electron microscopy.

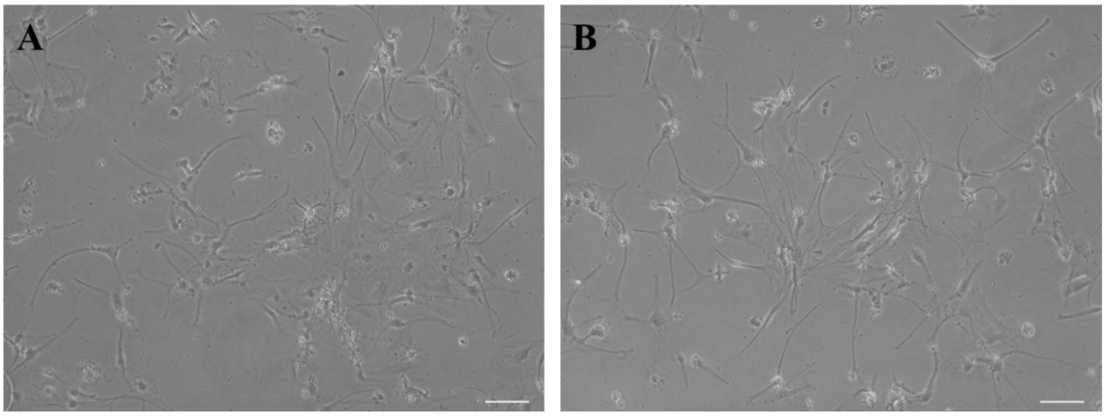

A. The morphological changes of primary cortical neurons were observed by electron microscopy before Compound C treatment.

B. Primary cortical neurons were treated with Compound C for 12 hours and the morphology of the cells was observed by electron microscopy.

Figure S10. Overexpression of HNF4α inhibited neuronal apoptosis after cardiac arrest.

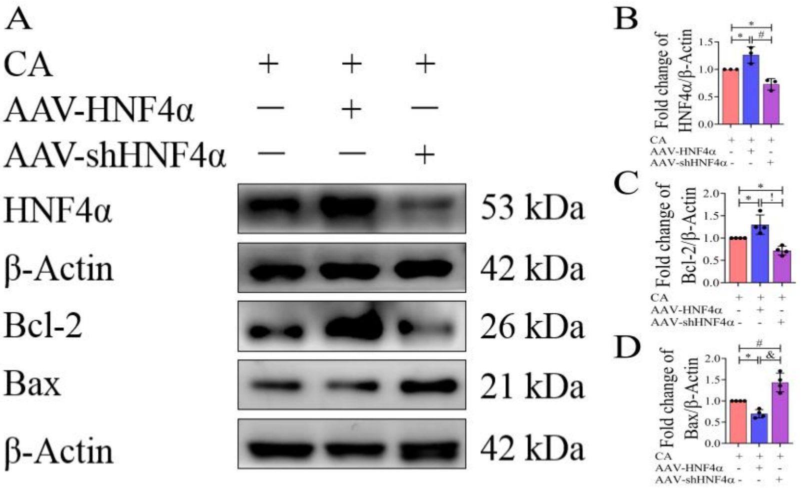

Figure S10. Effect of overexpression of HNF4α on apoptosis-related proteins.

A. The effect of HNF4α on apoptosis of hippocampal neurons were detected by western blot.

B-D. Statistical analysis of HNF4α and apoptosis-related proteins.
