# Supplementary material for: Targeted Activation of HNF4α by AMPK Inhibits Apoptosis and Ameliorates Neurological Injury Caused by Cardiac Arrest in Rats
Source: Neurochem Res. 2023 Jun 20;48(10):3129–45. doi: 10.1007/s11064-023-03957-1 (PMC10471732; doi:10.1007/s11064-023-03957-1)
Supplement: Supplementary file 2 — Supplementary file2 (DOCX 282 KB) [file 11064_2023_3957_MOESM2_ESM.docx]

**Table S1 The primer sequences of AMPK and HNF4α.**


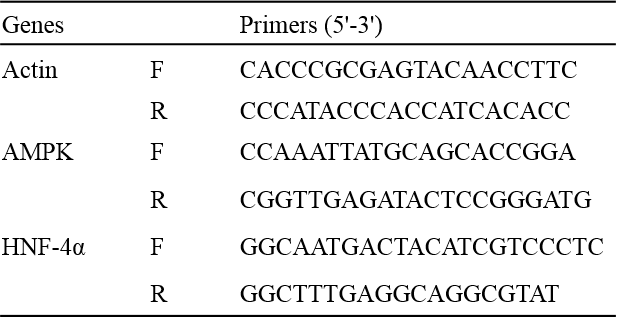


**Table S2 The first part of the animal basic physiological characteristics.**

**
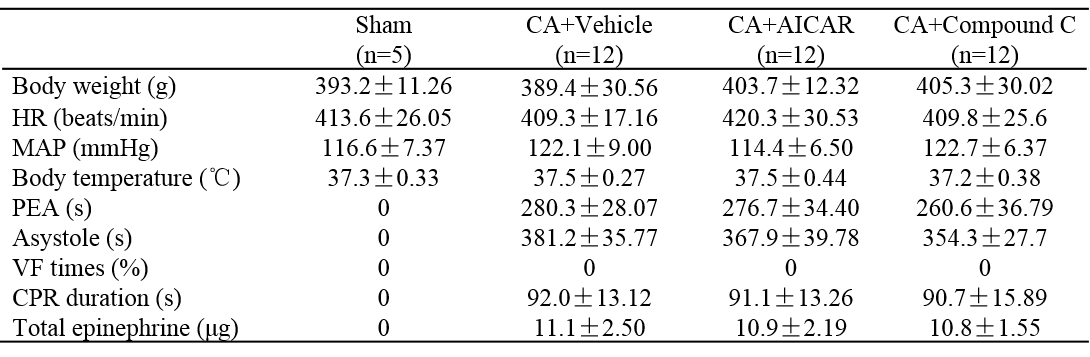
**

All values are presented as mean ± SD. There are no statistically significant differences between groups.

**Table S3 The second part of the animal basic physiological characteristics.**

**
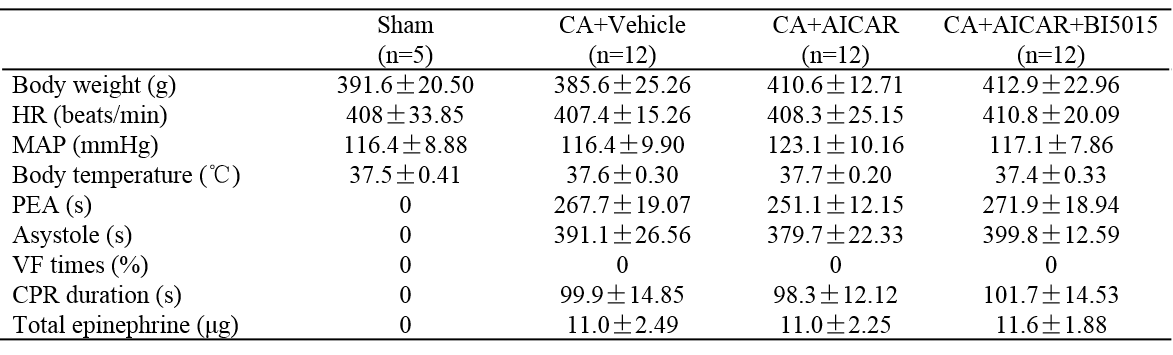
**

All values are presented as mean ± SD. There are no statistically significant differences between groups.

Abbreviations: CA: cardiac arrest, HR: heart rate, MAP: mean arterial pressure, PEA: pulseless electrical activity, MAP < 30 mmHg, Asystole: MAP < 10 mmHg, VF times: Ventricular Fibrillation times, CPR duration: cardiopulmonary resuscitation duration.
